# Immunogenicity of a DNA-Based Sindbis Replicon Expressing Crimean–Congo Hemorrhagic Fever Virus Nucleoprotein

**DOI:** 10.3390/vaccines9121491

**Published:** 2021-12-16

**Authors:** Thomas Tipih, Mark Heise, Felicity Jane Burt

**Affiliations:** 1 Division of Virology, University of the Free State, Bloemfontein 9300, South Africa; ttipih@gmail.com; 2 Department of Genetics, University of North Carolina at Chapel Hill, Chapel Hill, NC 27599, USA; mark_heisem@med.unc.edu; 3 Division of Virology, National Health Laboratory Service, Bloemfontein 9300, South Africa

**Keywords:** Crimean–Congo hemorrhagic fever virus, nucleoprotein, Sindbis virus replicon vector, immune response

## Abstract

Crimean–Congo hemorrhagic fever virus (CCHFV) infrequently causes hemorrhagic fever in humans with a case fatality rate of 30%. Currently, there is neither an internationally approved antiviral drug nor a vaccine against the virus. A replicon based on the Sindbis virus vector encoding the complete open reading frame of a CCHFV nucleoprotein from a South African isolate was prepared and investigated as a possible candidate vaccine. The transcription of CCHFV RNA and recombinant protein production by the replicon were characterized in transfected baby hamster kidney cells. A replicon encoding CCHFV nucleoprotein inserted in plasmid DNA, pSinCCHF-52S, directed transcription of CCHFV RNA in the transfected cells. NIH-III heterozygous mice immunized with pSinCCHF-52S generated CCHFV IgG specific antibodies with notably higher levels of IgG2a compared to IgG1. Splenocytes from mice immunized with pSinCCHF-52S secreted IFN-γ and IL-2, low levels of IL-6 or IL-10, and no IL-4. No specific cytokine production was registered in splenocytes of mock-immunized mice (*p* < 0.05). Thus, our study demonstrated the expression of CCHFV nucleoprotein by a Sindbis virus vector and its immunogenicity in mice. The spectrum of cytokine production and antibody profile indicated predominantly Th1-type of an anti-CCHFV immune response. Further studies in CCHFV-susceptible animals are necessary to determine whether the induced immune response is protective.

## 1. Introduction

Crimean–Congo hemorrhagic fever virus (CCHFV) exclusively causes disease in humans. The virus has an extensive global geographic distribution, and the disease has been reported in a wide array of countries in Africa, Asia, the Middle East, Eastern Europe and recently in Spain, Western Europe [1,2,3,4]. A total of seven human cases have been reported in Spain from 2016 to August 2020, with a case fatality rate of 42.9% [5]. Data from the Spanish studies indicate an establishment of the CCHFV transmission cycle in the country [5]. The expanding geographical range of the virus prompts intensification of the research and development efforts to prevent the disease, as for now, no internationally approved anti-CCHFV drugs or vaccines are available.

CCHFV is maintained in a life cycle involving ticks and vertebrate animals [1], while humans are regarded as incidental hosts. *Hyalomma* tick species serve as principal host and viral vectors [6]. Sources of human infections include infected tick bites, exposure to infected livestock blood or tissue, and human to human transmission, especially in hospital settings [7]. Infections are associated with the hemorrhagic syndrome, with a mortality rate of 30%.

CCHFV is classified in the *Nairoviridae* family and the *Orthonairovirus* genus. Structurally, Crimean–Congo hemorrhagic fever (CCHF) virions are spherical, measuring approximately 100 nm in diameter. The three single-stranded negative RNA segments are enclosed in a host cell-derived lipid bilayer membrane envelope. The RNA segments identified as small (S), medium (M), and large (L) each have a coding region sandwiched between 5′ and 3′ untranslated regions [8]. The untranslated regions present at the 5′ and 3′ termini of the S, M and L segments possesses sequences required for transcription, replication and packaging [8]. The S segment encodes the nucleocapsid protein (NP) and a non-structural S protein (NSs). The NP encapsidates viral RNA and complementary RNA [9], while the NSs demonstrates apoptotic activity in transfected cells [10]. The apoptotic roles of NSs are exclusively based on protein overexpression, thus warranting further investigation on NSs functions and properties [9]. The M segment directs the synthesis of a glycoprotein precursor, which is sequentially cleaved by host proteases in the endoplasmic reticulum and Golgi apparatus, to produce envelope glycoproteins (Gn and Gc), non-structural M protein, and secreted non-structural proteins [11,12,13]. The Gn–Gc heterodimer mediates viral assembly, budding of the newly formed virus particle, and attachment to new target cells [14]. The use of a well-established CCHFV virus-like particle system allowed researchers to demonstrate the relevance of each of the M-segment non-structural proteins to virus assembly, egress, and infectivity [15]. The L segment encodes for the RNA-dependent RNA polymerase. The L protein functions in viral mRNA transcription and translation [14]. As part of the viral RNA polymerase, CCHFV encodes a specific protease belonging to the ovarian tumor (OTU) superfamily, with both deubiquitinase and deISGylase activities, critical for the suppression of antiviral interferon response [16].

Internationally licensed therapies or vaccines against CCHFV are not yet available, thus treatment of CCHFV infection is mainly supportive. There is an inactivated vaccine used on a small scale in Eastern Europe [17]. It is unlikely that this inactivated vaccine may be licensed for international use because of safety reasons. The search for a CCHFV vaccine is thus an area of intense research. Vaccine candidates based on the inactivated CCHFV particle, the nucleoprotein, the glycoprotein precursor or the envelope glycoproteins have been reported [18,19,20,21,22,23,24]. The NP is the most abundantly produced viral protein during infection, is highly immunogenic, and possesses B and T cell epitopes [25,26]. However, unlike the envelope glycoproteins Gn and Gc, which are extracellular and induce neutralizing antibodies [27], the NP is an internal protein and does not induce neutralizing antibodies. Instead, the NP induces cytotoxic T lymphocyte response (CTL). Potent CTL responses have been demonstrated against the NP in survivors from CCHFV infection, whereas CTL response against structural glycoproteins is rare [26]. Importantly, anti-NP CTL responses are present at high frequency and are detectable several years after the acute infection, despite the absence of continued antigenic stimulation [26]. This advances the NP as a prospective candidate for the CCHFV vaccine.

Vaccine candidates based on the NP for orthohantaviruses and the Rift Valley fever virus have induced protective immune responses against lethal viral challenge in animal models [28,29]. Likewise, CCHF NP candidate vaccines have protected knockout mice in viral challenge studies [30,31], although negative results have also been reported [23]. Even though the NP is expected to induce a CTL response of protective character [32], as has been shown in the human studies [26], the immune correlates of the NP-induced protection in animal models remain unidentified [4].

Replicating viral vectors based on alphaviruses, flaviviruses, measles virus, and rhabdoviruses has been evaluated for vaccine development [33]. Sindbis virus, a member of the alphavirus genus, causes mild disease in humans, and replicons based on the Sindbis virus genome have demonstrated encouraging results as vaccine vectors [34]. Replication-deficient alphavirus vectors can be applied as naked RNA, recombinant particles, and plasmid DNA [35]. Recombinant particles are more effective at stimulating immune responses; however, the manufacturing process is costly, and safety issues arising from recombination generating replication-competent virus remain a concern. To circumvent these drawbacks, the RNA replicon can be launched from a plasmid. In this setup, the Sindbis virus genome sequence, excluding sequences encoding structural proteins, is converted to a cDNA sequence which is driven by a foreign promoter, such as one derived from human cytomegalovirus (hCMV). Transcription, beginning in the nucleus, results in the generation of the Sindbis virus RNA which exits the nucleus into the cytoplasm where the translation of the viral replicase takes place. The replicase directs transcription of subgenomic mRNA encoding the gene of interest [36,37]. Replicons are superior to conventional plasmid DNA vectors in terms of heterologous protein production because the Sindbis virus replication machinery generates numerous subgenomic mRNAs from which the gene of interest can be translated, in contrast to the traditional DNA vectors from which fewer mRNA transcripts are generated. Sindbis replicons promote apoptosis in transfected cells, and apoptosis enhances the induction of immune response [38,39]. Furthermore, Sindbis replicons are relatively safer, since apoptosis induced by the vector reduces the chances of foreign DNA integration. In this study, we present the characterization of a replicon expressing CCHFV NP in mammalian cells and evaluation of immune responses induced by this replicon delivered by plasmid DNA in a mouse model.

## 2. Materials and Methods

### 2.1. Cells

The baby hamster kidney (BHK-21) cell line (ATCC^®^ CCL-10, Manassas, VA, USA) was obtained from American Type Culture Collection (ATCC) and maintained in Dulbecco’s Modified Eagle Medium (DMEM), supplemented with 10% gamma-irradiated fetal bovine serum (FBS) (Gibco, Paisley, UK), 1% L-glutamine (Lonza, Verviers, Belgium), 1% penicillin-streptomycin (Lonza, Verviers, Belgium), and 1% non-essential amino acids (Lonza, Verviers, Belgium) at 37 °C in a humid 5% CO_2_ atmosphere.

### 2.2. Sindbis Replicon Vectors Expressing CCHFV NP

A primer set amplifying the entire open reading frame of the CCHFV nucleoprotein of CCHFV isolate SPU 187/90 was designed using sequence data retrieved from GenBank (GenBank Accession number: KJ682823.1).

Primers designated CCHF-NP-F: GCGGCCGCATGGAAAACAAAAT(T/C)GAGGTGAAT and CCHF-NP-R: ATCGATTTA*GTGGTGGTGGTGGTGGTG*GAT(G/A)ATGTT(G/A)GCACTGGTGGC were modified to include *Not*I and *Cla*I restriction enzyme sites for downstream cloning, and a polyhistidine tag (italicized) was included at the 3′ end of the reverse primer.

The primer pair F2 (TGGACACCTTCACAAACTC) and R3 (GACAAATTCCCTGCACCA) [40,41,42] that amplify a 536 base pair region of the nucleoprotein gene was used for the quantitative polymerase chain reaction (qPCR). A hydrolysis probe designated CCHF-NP31_52 (CTGAGCTAAAAGTTGACGTCCCGAAAAT) was identified, which annealed within the targeted region.

The pSinGFP expression vector encoding the green fluorescence protein (GFP) gene was provided by Professor Mark Heise from the University of North Carolina (Chapel Hill, NC, USA). CCHFV RNA was supplied by the National Institute for Communicable Diseases (Johannesburg, South Africa). Viral RNA was converted to cDNA using SuperScript^TM^ III Reverse Transcriptase (RT) enzyme (Invitrogen, Carlsbad, CA, USA) according to the manufacturer’s instructions. The CCHFV NP full-length open reading frame (from CCHFV isolate SPU 187/90, GenBank Accession number: KJ682823.1) was amplified using Phusion^®^ High fidelity (HF) DNA polymerase enzyme (New England Biolabs, Ipswich, MA, USA), using cDNA as a template in a two-step reverse transcription-polymerase chain reaction (RT-PCR) technique. The PCR cycling conditions comprised an initial denaturation at 98 °C for 30 s, followed by 25 cycles of 98 °C denaturation for 10 s, annealing at 68 °C for 30 s, and extension at 72 °C for 1.5 min followed by a final extension at 72 °C for 7 min. Samples were held at 4 °C. The amplified fragments of the nucleoprotein were purified and subcloned into an intermediate vector, pMiniT 2.0 (New England Biolabs, Ipswich, MA, USA), following the manufacturer’s instructions. Restriction enzyme digestion of the NP fragments in pMiniT 2.0 vector yielded DNA fragments that were inserted at the *Not*I and *Cla*I sites of the Sindbis-based replicon vector. Sanger sequencing was performed to determine the accuracy of the nucleotide sequences. Plasmids produced by transforming single-use JM109 competent cells (Promega, Madison, WI, USA) were purified using Qiagen Plasmid Maxi Kits (Qiagen, Hilden, Germany). The DNA concentration was determined using the NanoDrop 2000 spectrophotometer, and labelled aliquots were stored at −20 °C until use.

### 2.3. Recombinant Protein Expression and Detection

Transfection by electroporation was performed using the Neon^®^ Transfection System MPK5000 (Invitrogen, Carlsbad, CA, USA), according to the manufacturer’s instructions. Briefly, cells were dissociated, counted, washed, resuspended in 100 µL of Resuspension Buffer R (Invitrogen, Carlsbad, CA, USA), and mixed with 15 µg of the plasmid. Electroporation was performed at 1500 V, 3 pulses, and 10 ms pulse width, and cells were grown at 37 °C in a 5% carbon dioxide enriched environment. NP expression in transfected BHK-21 cells was detected using immunofluorescence microscopy. Briefly, 24 h post-transfection the cells were fixed in a methanol–acetone solution for 20 min at −20 °C and blocked with a solution consisting of 10% sucrose and 0.5% Triton X-100 in 1% phosphate-buffered saline (PBS) at room temperature for 20 min. Serum from CCHF survivors or anti-His_6_ mouse monoclonal antibody was applied 1:10 or 1:200, respectively. Detection involved goat anti-human and goat anti-mouse IgG fluorescein isothiocyanate antibody (FITC) (SeraCare Life Sciences, Milford, MA, USA), diluted 1:20 in 0.1% Evans blue. Cells were visualized using the Nikon ECLIPSE Ni-U fluorescence microscope (Melville, Huntington, NY, USA).

Transfected BHK-21 cells were grown in T-25 cm^2^ flasks at 37 °C in a 5% carbon dioxide enriched incubator for 48 h. Cells were washed and lysed in 250 µL of mammalian cell lysis buffer (50 mM Tris, 150 mM NaCl, 1% Nonidet P-40 with the addition of a protease inhibitor cocktail (Sigma, Burlington, MA, USA)). Harvested cells were separated using 12% sodium dodecyl sulfate-polyacrylamide gel (SDS-PAGE) electrophoresis and transferred onto polyvinylidene difluoride (PVDF) membrane. The PVDF membrane was probed with an anti-His_6_ monoclonal antibody (Roche, Rotkreuz, Switzerland), targeting the C-terminal histidine tag fused to the CCHFV nucleoprotein and detected using the Pierce^®^ Fast Western Blotting kit (Thermo Scientific, Waltham, MA, USA). The images were captured with the C-DiGit^®^ Blot Scanner (LI-COR^®^, Bad Homburg, Germany).

### 2.4. Quantitative Determination of CCHFV NP RNA

Transfected BHK-21 cells grown in 6-well plates were harvested at 4 h, 8 h, 12 h, 24 h, and 48 h. Total RNA was extracted using the RNeasy^®^ Plus Mini Kit (Qiagen, Hilden, Germany) as per the manufacturer’s instructions. DNA removal from RNA samples was performed using the RQ1 RNase-free DNase kit (Promega, Madison, WI, USA) according to the manufacturer’s instructions.

Quantitative real-time PCR was performed using the LightCycler^®^ 2.0 Instrument (Roche, Rotkreuz, Switzerland). The master mix was prepared by adding 10 µL of the LightCycler^®^ FastStart Enzyme to the LightCycler^®^ FastStart TaqMan^®^ Reaction Mix containing reaction buffer, MgCl_2_, and dNTP mix, all supplied with the kit. The reactions consisted of 4 µL master mix, 2 µL (10 pmol/µL) each of F2 and R3, 1 µL (2 pmol/µL) CCHF-NP31_52 (hydrolysis probe), 1 µL cDNA/diluted standards, and 10 µL nuclease-free water. Thermocycling conditions comprised of a pre-incubation step at 95 °C, followed by 35 cycles of denaturation at 95 °C for 10 s, annealing and extension at 55 °C for 60 s, and a cooling step at 40 °C for 30 s. The fluorescence signal was gathered at the end of the combined annealing and extension step of each cycle. A temperature transition rate of 20 °C/s was utilized. Samples were analyzed in duplicate, and each run included a known standard. The concentrations of samples were determined using standard curves generated from a positive control of known copy numbers.

### 2.5. Animal Immunizations

Six- to eight-week-old female National Institute of Health (NIH), NIH-III Heterozygous mice strain were bred and housed in the animal facility at the University of the Free State. Mice in groups of three were immunized with either 100 µg of Sindbis replicon expressing CCHFV NP, or 100 µg of the replicon and 50 µg of Polyinosinic-polycytidylic acid Poly (I:C) HMW (High Molecular Weight) VacciGrade (InvivoGen, Toulouse, France) into the tibia anterior muscle at days 0, 21, and 42. The control group received 50 µg of pSinGFP and 50 µg of Poly (I:C) HMW VacciGrade. The replicon and the adjuvant were administered in a total volume of 100 µL, 50 µL in each of the tibia anterior muscle. Mice were monitored daily for any signs of discomfort post-vaccination.

### 2.6. Determination of Cytokine Responses

To evaluate cytokine secretion as an index of cellular immune responses, mice spleens were stimulated in vitro using CCHFV antigen. The spleens were harvested from mice euthanized 2 weeks after the final immunization. Spleens were harvested from euthanized mice and single-cell suspensions prepared in RPMI 1640 media containing 10% heat-inactivated foetal bovine serum. Single-cell suspensions were seeded at a density of 5 × 10^5^ cells per well in 96 well plates, in a total volume of 250 µL RPMI 1640 media supplemented with 10% gamma-irradiated foetal bovine serum and stimulated with 10 µg of inactivated sucrose acetone extracted CCHFV antigen prepared from infected suckling mouse tissue for 48 h at 37 °C in a 5% CO_2_ enriched incubator. The antigen was prepared by intracranial inoculation of suckling mice with a South African isolate of CCHFV (SPU 4/81) and extraction of antigen from brain tissue of mice that succumbed [43] (kindly donated by Prof J Paweska from the National Institute for Communicable Diseases, South Africa). Meanwhile, 0.25 µg/well of concanavalin A antigen (Calbiochem, San Diego, CA, USA) and PBS were used as positive and negative controls respectively. The CCHFV antigen was supplied freeze-dried and reconstituted in sterile PBS prior to stimulating splenocytes. Stimulation was performed in duplicate for each of the cytokine assayed.

The levels of interleukin (IL)-2, IL-4, IL-6, IL-10, tumor necrosis factor-alpha (TNF-α), and interferon-gamma (IFN-γ) in culture supernatants were determined by ELISA kits (eBioscience, San Diego, CA, USA) following manufacturer’s instructions. Photometric analysis was performed at 450 nm using the Sunrise™ absorbance reader. All assays were performed in duplicate.

### 2.7. Determination of Humoral Immune Responses

CCHFV NP-specific immunoglobulins G (IgG) were analyzed in serum from immunized mice using an indirect immunofluorescent assay (EUROIMMUN AG, Lubeck, Germany), modified to test mouse serum samples. The assay was supplied with BIOCHIP slides coated with cells expressing the CCHFV nucleoprotein. Sera from a laboratory confirmed human CCHF survivor previously shown to have antibody was used as a positive control. Serum samples were reacted with cells on the BIOCHIP at room temperature. After washing with PBS-Tween, the slides were incubated with fluorescein-labelled goat anti-mouse IgG (SeraCare Life Sciences, Milford, MA, USA), diluted 1:20 in 0.1% Evans blue for 30 min at room temperature. Slides were visualized using the Nikon ECLIPSE Ni-U fluorescence microscope. Anti-CCHFV NP positive samples were further isotyped to determine the IgG subtypes using fluorescein-conjugated rat anti-mouse IgG1, IgG2a, and IgG2b (Biolegend, San Diego, CA, USA) as detection antibodies.

### 2.8. Statistical Analysis

Statistical analysis (Mann–Whitney test) was performed using GraphPad Prism version 9.2.0 for Windows (GraphPad Software, San Diego, CA, USA, www.graphpad.com, 10 December 2021). Statistical significance was set at *p* < 0.05.

## 3. Results

### 3.1. Expression of CCHFV NP by Sindbis Replicon Vector

**Figure 1:**
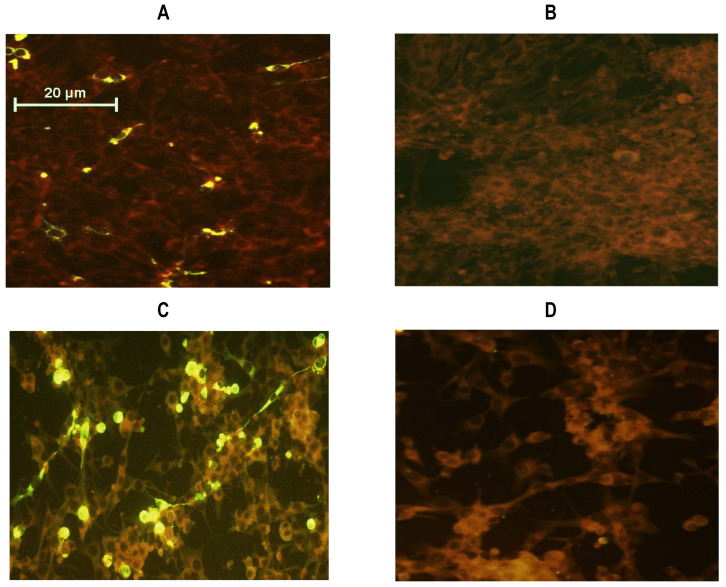

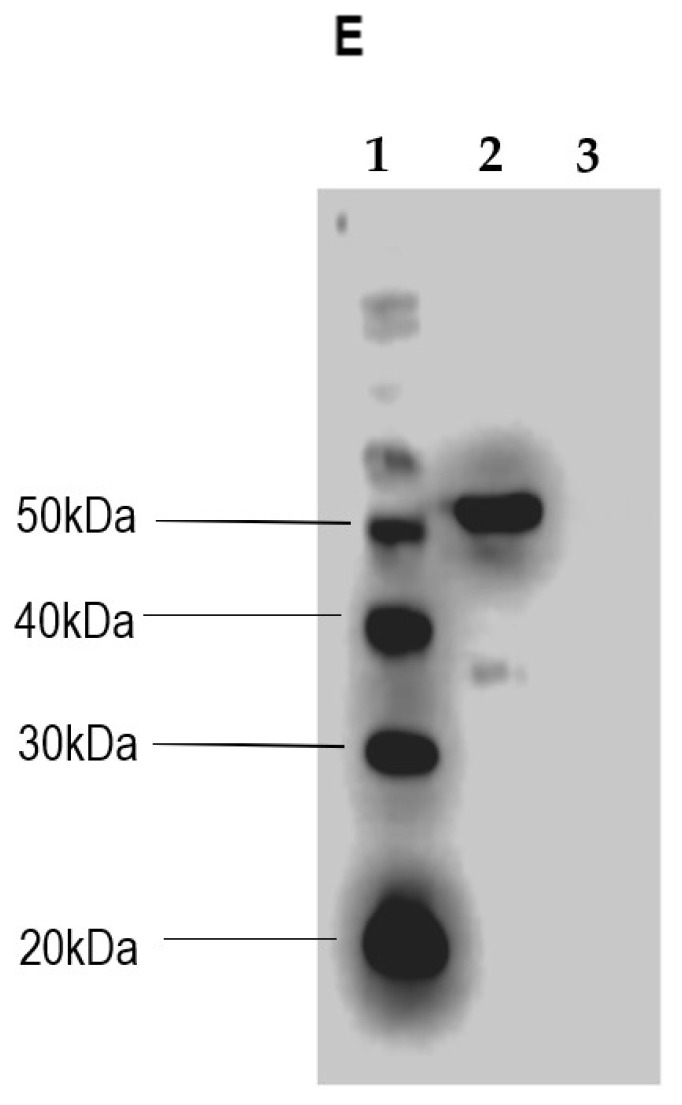
BHK-21 cells transfected with pSinCCHF-52S encoding His-tagged CCHFV nucleoprotein (NP) fused to the polyhistidine tag were specifically stained with anti-His_6_ antibody (**A**,**B**) and anti-CCHFV IgG human serum (**C**,**D**) in immunofluorescence test, and expressed protein with a molecular mass of 52 kDa stained with anti-His tag antibodies (**E**). BHK-21 cells transfected with replicon pSinCCHF-52S (**A**,**C**); mock-transfected BHK-21 cells (**B**,**D**); images were captured using the Olympus BX51 fluorescence microscope (USA) (×40). Western-blot analysis of CCHFV NP using mouse anti-His6 monoclonal antibody (**E**): Lane 1: MagicMark XP Western Protein Standard, Lane 2: BHK-21 cells transfected with pSinCCHF-52S, Lane 3: Mock-transfected BHK-21 cells. The position of the molecular mass marker is shown on the left.

CCHFV NP gene was amplified from a South African viral isolate by the RT-PCR technique. Translated NP amino acid sequence showed 100% similarity compared to the original CCHFV NP sequences deposited in the GenBank. The CCHFV NP encoding sequence was cloned into a replicon based on the Sindbis virus vector generating recombinant plasmid, designated pSinCCHF-52S (Supplementary Figure S1). The NP sequence was supplemented with a polyhistidine tag sequence at the C-terminal. CCHFV nucleoprotein expression in BHK-21 cells transfected with the recombinant plasmid, pSinCCHF-52S, was evaluated by immunofluorescence staining of expressing cells using anti-His tag antibodies (Figure 1A,B) and further confirmed by immunofluorescence staining using anti-CCHF IgG from CCHF survivors (Figure 1C,D). SDS-PAGE, followed by transfer to the PVDF membrane and Western blotting with anti-His_6_ tag mouse monoclonal antibody, revealed a protein band of approximately 52 kDa corresponding to the estimated molecular mass of CCHFV NP [44] (Figure 1E and Figure S5).

### 3.2. Kinetics of CCHF NP Total RNA Expression

The kinetics of CCHFV NP RNA transcription by pSinCCHF-52S in transfected BHK-21 cells was evaluated by performing a qRT-PCR. For this, total RNA was extracted from the transfected BHK-21 cells at 4, 8, 12, 24, and 48 h post-transfection. Total RNA was converted to cDNA, and cDNA was quantified as a proxy for the original subgenomic CCHFV NP RNA by the RT-qPCR technique. The CCHFV NP RNA load was estimated using a standard curve generated by ten-fold dilution, ranging from 1.28 × 10^11^ to 1.28 × 10^3^ DNA copies (amplification efficiency of 1.928 with a standard error of 0.018).

**Figure 2:**
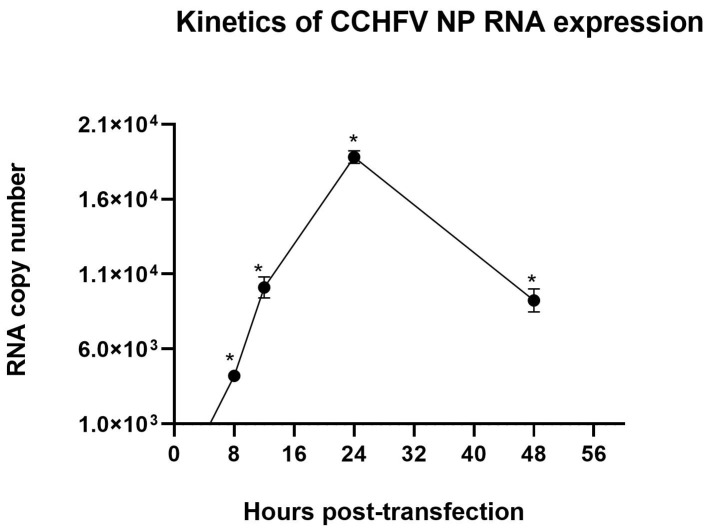
Kinetics of CCHFV NP total RNA expression in BHK-21 cells electroporated with pSinCCHF-52S registered 4 to 48 h post-transfection. No CCHFV RNA was detected in cells after transfection with pSinGFP. * Signal was significantly higher than that generated by qRT-PCR of cells transfected with pSinGFP (<1.0 × 10^3^; *p* < 0.05; Supplementary Table S1).

qRT-PCR demonstrated that transcription of CCHFV NP RNA from pSinCCHF-52S construct started 8 h post-transfection, increasing sharply by 24 h and decreasing thereafter (Figure 2). No CCHFV RNA was detected in cells transfected with a plasmid carrying Sindbis virus replicon expressing GFP (pSinGFP). Copy numbers of NP RNA detected 4 to 48 h post-transfection are presented in Supplementary Table S1.

### 3.3. Immune Responses of Mice to Immunization with pSinCCHF-52S

Plasmid pSinCCHF-52S expressing CCHFV NP was used to immunize NIH mice (*n* = 5); two of these mice received pSinCCHF-52S mixed with Poly (I:C). Control mice received pSinGFP mixed with Poly (I:C). Plasmids were delivered by intramuscular injections on days 0, 21, and 42. At the experimental endpoint, mice were bled, sera were collected, thereafter mice were euthanized, and spleens were excised to prepare single-cell splenocyte cultures for T-cell tests.

#### 3.3.1. Humoral Immune Response

**Figure 3:**
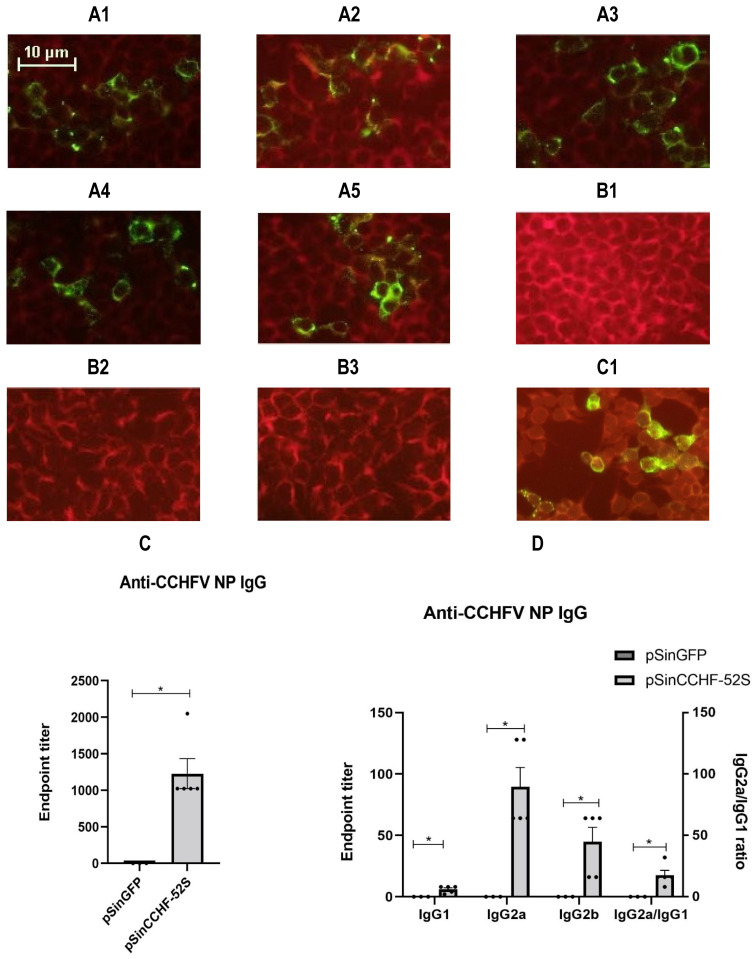
Antibody responses induced by immunization with pSinCCHF-52S. Indirect immunofluorescence analysis of serum CCHFV NP IgG antibodies from pSinCCHF-52S immunized mice with and without Poly (I:C) (**A1**–**A5**): (**A1**) mouse 1, (**A2**) mouse 2, (**A3**) mouse 3, (**A4**) mouse 4, (**A5**) mouse 5 compared to mice immunized with pSinGFP (**B1**–**B3**): (**B1**) mouse 1, (**B2**) mouse 2, and (**B3**) mouse 3. (**C1**)**,** positive control. Images were captured using the Nikon ECLIPSE Ni-U fluorescence microscope (USA) (×20). Anti-CCHFV NP IgG endpoint titer (**C**). Anti-CCHFV NP IgG endpoint titer and IgG2a/IgG1 ratios (**D**). Mice (NIH; *n* = 5/group) were immunized three times intramuscularly with the prepared pSinCCHF-52S construct expressing CCHFV nucleoprotein with (*n* = 2) and without Poly (I:C) (*n* = 3). Serum anti-CCHFV NP IgG were analyzed using a commercially available indirect immunofluorescent assay. Data are expressed as the mean for five mice (pSinCCHF-52S) and three mice for pSinGFP and the standard error of the mean. * *p* < 0.05 by Mann–Whitney U test.

Plasmid pSinCCHF-52S induced the production of CCHFV nucleoprotein-specific antibodies (Figure 3A1–A5). No antibodies were detected in mice receiving control pSinGFP plasmid (Figure 3B1–B3). The average endpoint titer of anti-NP antibodies determined by a commercial indirect immunofluorescent assay reached 1229 ± 457.9 (Figure 3C).

Anti-nucleoprotein NP antibodies were further characterized into the IgG1, IgG2a, and IgG2b subtypes (Figure 3D). Subtyping demonstrated higher endpoint titers of anti-NP IgG2 subtypes (Figure 3D, Supplementary Figure S2B). The ratios of IgG2a/IgG1 and of IgG2a/IgG2b exceeded 1 (Supplementary Figure S2B). The addition of Poly (I:C) led to a decrease in the titers of anti-NP IgG, IgG1, IgG2a, and IgG2b; however, the difference did not reach the level of significance and had no effect on the IgG2a/IgG1 and IgG2a/IgG2b ratios (Supplementary Figure S2B).

#### 3.3.2. Cellular Immune Response

**Figure 4:**
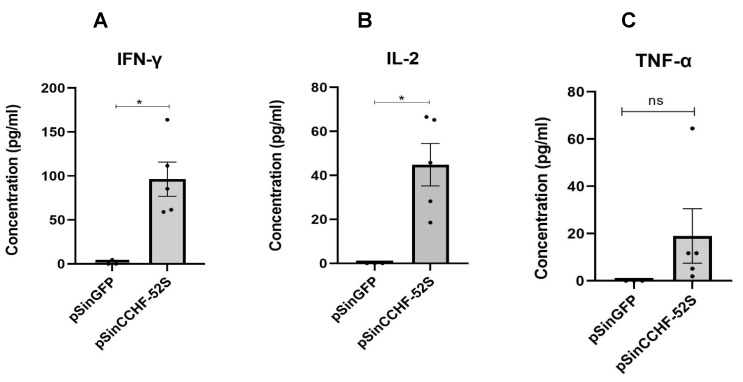
Immunization of mice with pSinCCHF-52S expressing NP protein of CCHFV (*n* = 5) induced specific production of IFN-γ (*p* = 0.0369) (**A**), IL-2 (*p* = 0.0495) (**B**), and an insignificant increase in production of TNF-a (*p* = 0.1) (**C**) as compared to cytokine responses in mice immunized with pSinGFP (*n* = 3). Murine splenocytes were stimulated with CCHFV antigen, as described in the Materials and Methods section. Cytokine expression was measured by ELISA using commercial kits (eBioscience, San Diego, CA, USA). Data are expressed as the mean for the group, with the standard error of the mean. * *p* < 0.05.

To investigate cytokine responses following immunization with Sindbis replicons expressing CCHFV NP with and without Poly (I:C), splenocytes harvested from vaccinated mice were stimulated in vitro with CCHFV antigen for two days, sterile PBS as the negative control or concanavalin A as the positive control. We performed a Mann–Whitney test on the levels of cytokines secreted by splenocytes from pSinCCHF-52S immunized mice with and without Poly (I:C). There was no statistically significant difference in the levels of IFN-γ and IL-2 production in mice receiving pSinCCHF-52S with or without Poly (I:C) (Supplementary Figure S3A,B). Thus, Poly (I:C) had no significant effect on cellular response. Splenocytes of mice immunized with pSinCCHF-52S secreted significantly higher levels of IFN-γ (*p* = 0.0357) and IL-2 (*p* = 0.0357), and insignificantly higher levels of TNF-α (*p* = 0.1000) compared to control mice immunized with pSinGFP (Figure 4A–C). Of note, TNF-α secretion by cells from mice that received the replicon in the absence of Poly (I:C) was marginally higher than those from the control group (*p* = 0.0495; Supplementary Figure S3C). Immunization with pSinCCHF-52S induced weak production of IL-6 and IL-10 (Supplementary Figure S4A,B). IL-4 was undetectable. These results demonstrate that immunization with pSinCCHF-52S expressing CCHFV NP protein induced predominantly Th1 type cytokines, indicating a Th1-profile of cellular immune response.

#### 3.3.3. Correlation between Cytokine and Antibody Responses

We have also looked for correlations between cellular and antibody responses to CCHFV NP using the Spearman rank correlation test. Considering that we had data for five immunized and three control mice, the test was run at a stringent significance value of *p* < 0.001. Strong correlations were observed between CCHFV antigen-specific splenocyte production of IFN-γ and TNF-α (R = 0.9326), and IL-2 and TNF-a (R = 0.957), confirming specificity of TNF-α production (Supplementary Table S2). Correlations indirectly indicated that these three cytokines were produced by the same T-cells.

Furthermore, both IFN-γ and IL-2 production correlated with anti-CCHFV NP IgG2a (R = 0.9506 for both; Supplementary Table S2). Induction of anti-CCHFV NP IgG1 and IgG2b was, on the contrary (and as could be expected) correlated with the levels of IL-6 (R = 0.9512 and 0.9449, respectively). Last but not least, the production of anti-CCHFV NP IgG1, IgG2a, and IgG2b was strongly correlated with the secretion of TNF-α (R = 0.974679, 0.968246, and 0.968246, respectively; Supplementary Table S2). Altogether, this confirmed Th-1 tilting of anti-CCHFV immune response in pSinCCHF-52S immunized mice.

## 4. Discussion

In the present study, we reported the preparation of a Sindbis replicon expressing the full-length open reading frame of a CCHFV NP from a South African isolate. The replicon based on a DNA vector transcribed CCHFV NP subgenomic RNA. We assessed the transcription of CCHFV NP RNA in the transfected BHK-21 cells by the qRT-PCR technique by introducing an index of self-replication. This allowed us to quantify the rate of self-replication of the Sindbis virus replicon expressing the CCHFV NP, which, according to our knowledge, has not previously been assessed. Self-replication was vital in underscoring the prepared recombinant plasmid as an efficient vector for immunogenicity studies. Another necessary attribute of the vector for immunization was its ability to direct the translation of the heterologous protein. Our experiments demonstrated the capacity of the Sindbis replicons to direct the expression of the CCHFV NP in vitro. An efficient self-replication of the Sindbis virus replicon made the pSinCCHF-52S construct potentially effective as an immunogen. An active alphavirus virus replicase offered an additional bonus: replicase activity produces dsRNA intermediates that activate antiviral pathways, thus potentiating the induction of immune responses [45].

Indeed, when introduced into mice, the construct pSinCCHF-52 induced nucleoprotein-specific antibodies and potent in vitro CCHFV specific cytokine production by murine splenocytes stimulated with CCHFV antigen, as a readout vaccine-induced cellular immunity. Memory splenocytes generated after the initial encounter with an antigen secrete cytokines upon re-exposure to the same antigen. We therefore stimulated and analyzed cytokine secretion by splenocytes harvested from immunized compared to control mock-immunized mice and observed specific cytokine secretion by splenocytes from vaccinated mice after stimulation by the CCHFV antigen. Predominantly produced were IFN-γ and IL-2; mice receiving pSinCCHF-52 without Poly (I:C) also produced TNF-α, while levels of IL-6 and IL-10 were low (not exceeding those in mice mock-immunized with pSinGFP and no IL-4). The profile of cytokines indicated a Th1-type immune response [46,47]. In a previous study by Aligholipour Farzani et al., the researchers reported high levels of serum IL-6 and TNF-α, which were postulated to be associated with survival in mice lacking both the IFN-I receptor and IFN-gamma receptor (IFNα/β/γR−/−) after immunization with NP based constructs [31]. Future studies are planned to assess the protective potential of the immune responses induced by the replicon and to evaluate serum cytokines that can be considered protective.

Immunization of mice with pSinCCHF-52S construct elicited CCHFV NP-specific IgG antibodies. CCHFV specific antibodies were of predominantly IgG2a subclass; the high IgG2a/IgGa and IgG2a/IgG2b ratios spoke of a predominantly Th1-type immune response [48,49]. The Th-1 immune response was also supported by strong correlations between anti-CCHFV NP IgG2a and CCHFV NP-specific IFN-γ and IL-2 production. However, the presence of both IgG2a and IgG1 in immunized mice suggests pSinCCHF-52S has the potential to elicit both Th1 and Th2 responses. Unlike our study, a previous study reported a bias towards Th2 responses in BALB/c mice following immunisation with constructs expressing the nucleocapsid protein from a CCHF Ank-2 strain [31]. We could not assess the neutralization potential of the NP-specific antibodies because of the absence of the Biosafety Level (BSL)-4 facility, but we did not expect this immunization to produce neutralizing antibodies, as neutralizing antibodies are normally observed after immunization with the CCHFV glycoproteins [27,50,51].

We observed strong correlations between CCHFV NP-specific IFN-γ, IL-2, and TNF-α production, and levels of anti-NP IgG2a antibodies. Strong correlations between antigen-specific IFN-γ, IL-2, and TNF-α production alongside the preferentially Th1-type immune response determined by antibody profiling were earlier shown in mice immunized with plasmid DNA encoding HIV-1 reverse transcriptase (RT) [52]. Specific responses were later attributed to the RT-specific T cell reactivity of CD4^+^ T cells [53]. Our data on the correlations between NP-specific IFN-γ, IL-2, and TNF-α production, together with these observations, speak in favor of all three cytokines being produced by the same cells, suggestively, NP-specific CD4^+^ T cells. Same cells could then give support to the production of NP-specific IgG2a.

Traditionally, adjuvants are incorporated into vaccines to increase the magnitude of antibody response or the potential to prevent infection and to guide the type of adaptive immune response, as per the immune correlates of protection [54]. Poly (I:C), a Toll-like receptor (TLR) agonist, was investigated as a potential adjuvant for the replicon. TLRs expressed by numerous innate immune cells recognize conserved molecular products on various pathogens, setting off a chain of signaling events resulting in activation of innate immunity and subsequent initiation of adaptive immunity [55]. Poly (I:C) exerts its adjuvant effects by activating TLR3 and MDA5 pathways, promoting the induction of antibody responses, Th1, and CD8^+^ T cells immune responses [54]. On the other hand, DNA vaccination generally promotes Th1 responses, which have been reported as effective against viruses in animal models [56]. Importantly, a dominant Th1 response has been suggested to confer the most efficient protective immune responses against lethal CCHFV challenge in a mouse model lacking type 1 interferon signalling (IFNAR^−/−^) [20]. A previous study reported CTL responses against the CCHFV NP from survivors [26], probably pointing to the importance of these immune responses in clearing the infection. We, therefore, sought to maximize Th1 immune responses by the replicon, thus selecting Poly (I:C) to serve as an adjuvant. However, mice co-vaccinated with pSinCCHF-52S and Poly (I:C) did not show an enhanced Th1 type response; instead, the adjuvant somewhat dampened both cellular and antibody responses. Given the possibility that Poly (I:C) is likely to exert its effects first before the transfected cells fully express the protein, by signaling type 1 interferon production, Poly (I:C) might have induced an antiviral state in cells, thus hindering Sindbis virus replicase activity and ultimately reducing transcription and translation of the encoded CCHFV NP. In support of this, innate immune responses induced in a human cell line pretreated with Poly (I:C) were shown to inhibit replication of the Chikungunya virus in vitro [57]. The possibility of overstimulation of the innate immune system by both the Sindbis virus vector and the Poly (I:C) subsequently interfering with the potency of the Sindbis replicon also merits consideration. Both Poly (I:C) and alphavirus-based vaccines induce antiviral pathways [39,54], which downregulate mRNA translation, thereby inhibiting protein synthesis [58,59,60]. Our results suggest that Poly (I:C) may not be the best adjuvant for viral vectors. In fact, adjuvants that interfere with viral replication dampen immune induction with viral vectors. However, the “side effects” of Poly (I:C) can be circumvented by administering Poly (I:C) at least 2–3 days following the introduction of the replicon, after which the expression of the CCHFV NP would have occurred. We intend to further investigate adjuvants that can enhance the immunogenicity of the CCHFV NP encoded by the Sindbis replicon. Plasmid encoded adjuvants would be ideal, since the immune stimulation and protein expression are synchronised. The genetic adjuvants can be administered as separate plasmids or as additional genes encoded by the replicon.

The present study has a number of limitations. A small number of mice were used in our study; consequently, a larger mouse sample size may be required to further validate the findings. We understand that harvested splenocytes consist of T lymphocytes (CD4^+^ and CD8^+^ T cells) among other white cells. The observed cytokine responses could have been either CD4^+^ and/or CD8^+^ T cells, and further studies are needed to identify their origins. Poly (I:C), intended to amplify cellular responses, specifically the Th1-type immune response, failed to play this role, possibly due to its interference with Sindbis replicon replication, or due to overstimulation of the immune system in addition to the one caused by RNA expressed by the Sindbis viral replicase.

Despite the limitations, CCHFV NP transcription in transfected BHK cells coupled with the cellular and humoral immune responses generated in pSinCCHF-52S immunized mice is encouraging. In continuation, we are planning to further enhance the immunogenicity of the NP in this vector system by including a signal peptide that can be incorporated into the N-terminal of the NP gene to promote the dissemination of the NP beyond the DNA transfected cells, thus providing broad access to the cells of the immune system. The nature of immune responses generated by combining either the CCHFV GPC/Gn and Gc with the NP warrant investigation. In summary, we have demonstrated that Sindbis-based replicons efficiently expressed the CCHFV NP, and construct pSinCCHF-52S induced predominantly Th1 immune responses in an animal model. Further studies in CCHFV animal models are necessary to determine whether the immune responses are protective of the lethal virus challenge.

## Data Availability

Data available upon request.

## Supplementary Materials

The following are available online at https://www.mdpi.com/article/10.3390/vaccines9121491/s1, Table S1: RT-qPCR viral load results following the transfection of pSinCCHF-52S in BHK-21 cells. Samples were analyzed in duplicate; Table S2: Spearman Rank Order Correlations. MD pairwise deleted. Correlations in bold are significant at *p* < 0.001, Figure S1: Preparation of recombinant plasmids. Schematic representation of pSinCCHF-52S; Figure S2: Antibody responses induced by immunization with pSinCCHF-52S with and without Poly (I:C); Figure S3: Cytokine profiling by ELISA from splenocytes harvested from NIH mice after immunization with pSinCCHF-52S independently (*n* = 3) or with adjuvant poly (I:C) (*n* = 2); Figure S4: Cytokine profiling by ELISA from splenocytes harvested from NIH mice (*n* = 5/group) after immunization with pSinCCHF-52S independently or with adjuvant poly (I:C); Figure S5: Western blot analysis of CCHFV NP.
