# Supplementary material for: Immunogenicity of a DNA-Based Sindbis Replicon Expressing Crimean–Congo Hemorrhagic Fever Virus Nucleoprotein
Source: Vaccines (Basel). 2021 Dec 16;9(12):1491. doi: 10.3390/vaccines9121491 (PMC8703447; doi:10.3390/vaccines9121491)
Supplement: Supplementary file 1 [file vaccines-09-01491-s001.zip › vaccines-1436368-supplementary.pdf]

Supplementary Materials

Table S1: RT-qPCR viral load results following the transfection of pSinCCHF-52S in BHK-21 cells. Samples were analyzed in duplicate.

Time after electroporation	Ct value	Estimated RNA copy number	Average RNA copy number
4 hrs<br>4 hrs	28.52<br>28.68	*NE	*NE
8 hrs<br>8 hrs	24.31<br>24.21	4.09 E3<br>4.31 E3	4.23 E3
12 hrs<br>12 hrs	22.86<br>23.01	1.06 E4<br>9.6 E3	9.65 E3
24 hrs<br>24 hrs	22.01<br>21.96	1.85 E4<br>1.91 E4	1.88 E4
48 hrs<br>48 hrs	22.98<br>23.16	9.78 E3<br>8.7 E3	9.24 E3

*NE – CCHFV RNA copy numbers not estimated

Table S2: Spearman Rank Order Correlations. MD pairwise deleted. Correlations in bold are significant at $p < 0.001$

**Variable**	**Anti-52S IgG1**	**Anti-52S IgG2a**	**Anti-52S IgG2b**	**Anti-52S IgG2a/IgG1**	**Anti-52S IgG2a/IgG2b**	**IFN-γ**	**IL-2**	**TNF-α**	**IL-10**	**IL-6**
Anti-52S IgG1	1.000000	**0.927173**	**0.993399**	-0.750000	-0.883883	0.906539	0.906539	**0.974679**	0.468692	**0.951190**
Anti-52S IgG2a	**0.927173**	1.000000	**0.933333**	0.000000	-0.304290	**0.950586**	**0.950586**	**0.968246**	0.363419	0.818923
Anti-52S IgG2b	**0.993399**	**0.933333**	1.000000	-0.645497	-0.912871	0.887214	0.887214	**0.968246**	0.446304	**0.944911**
Anti-52S IgG2a/IgG1	-0.750000	0.000000	-0.645497	1.000000	0.824958	-0.223607	-0.223607	-0.447214	-0.223607	-0.894427
Anti-52S IgG2a/IgG2b	-0.883883	-0.304290	-0.912871	0.824958	1.000000	-0.263523	-0.263523	-0.632456	0.263523	-0.948683
IFN-γ	0.906539	**0.950586**	0.887214	-0.223607	-0.263523	1.000000	0.921687	**0.932673**	0.448493	0.790433
IL-2	0.906539	**0.950586**	0.887214	-0.223607	-0.263523	0.921687	1.000000	**0.957217**	0.375764	0.778457
TNF-α	**0.974679**	**0.968246**	**0.968246**	-0.447214	-0.632456	**0.932673**	**0.957217**	1.000000	0.395092	0.902708
IL-10	0.468692	0.363419	0.446304	-0.223607	0.263523	0.448493	0.375764	0.395092	1.000000	0.542208
IL-6	**0.951190**	0.818923	**0.944911**	-0.894427	-0.948683	0.790433	0.778457	0.902708	0.542208	1.000000

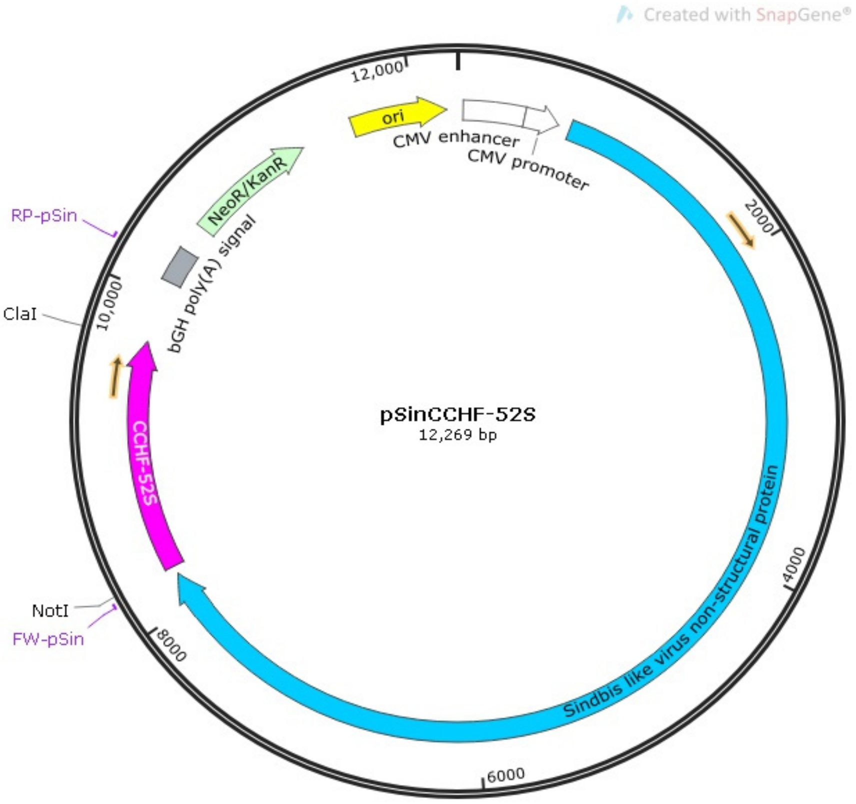

Figure S1. Preparation of recombinant plasmids. Schematic representation of pSinCCHF-52S.

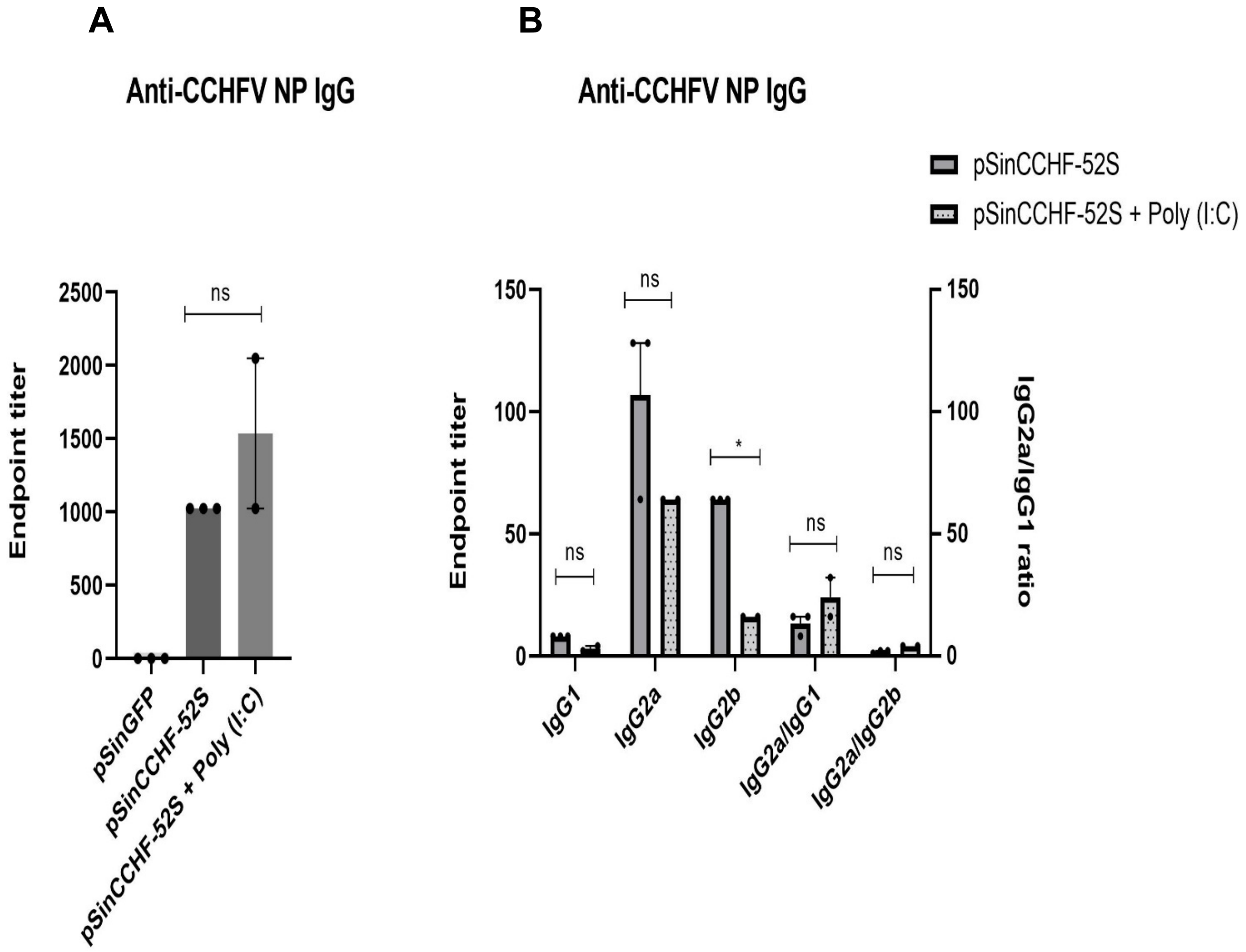

Figure S2. Antibody responses induced by immunization with pSinCCHF-52S with and without Poly (I:C). **(A)** Total anti-CCHFV NP endpoint titer **(B)** Anti-CCHFV NP IgG isotypes endpoint titer. Mice (NIH; n = 3/group) were immunized three times intramuscularly with the prepared pSinCCHF-52S construct expressing CCHFV nucleoprotein with and without Poly (I:C). Serum anti-CCHFV NP IgG subtypes were analyzed using an indirect immunofluorescent assay. Data are expressed as the mean for three mice (pSinCCHF-52S) and two mice for pSinCCHF-52S+Poly (I:C) and the standard error of the mean. * Statistical analysis performed using the F test.

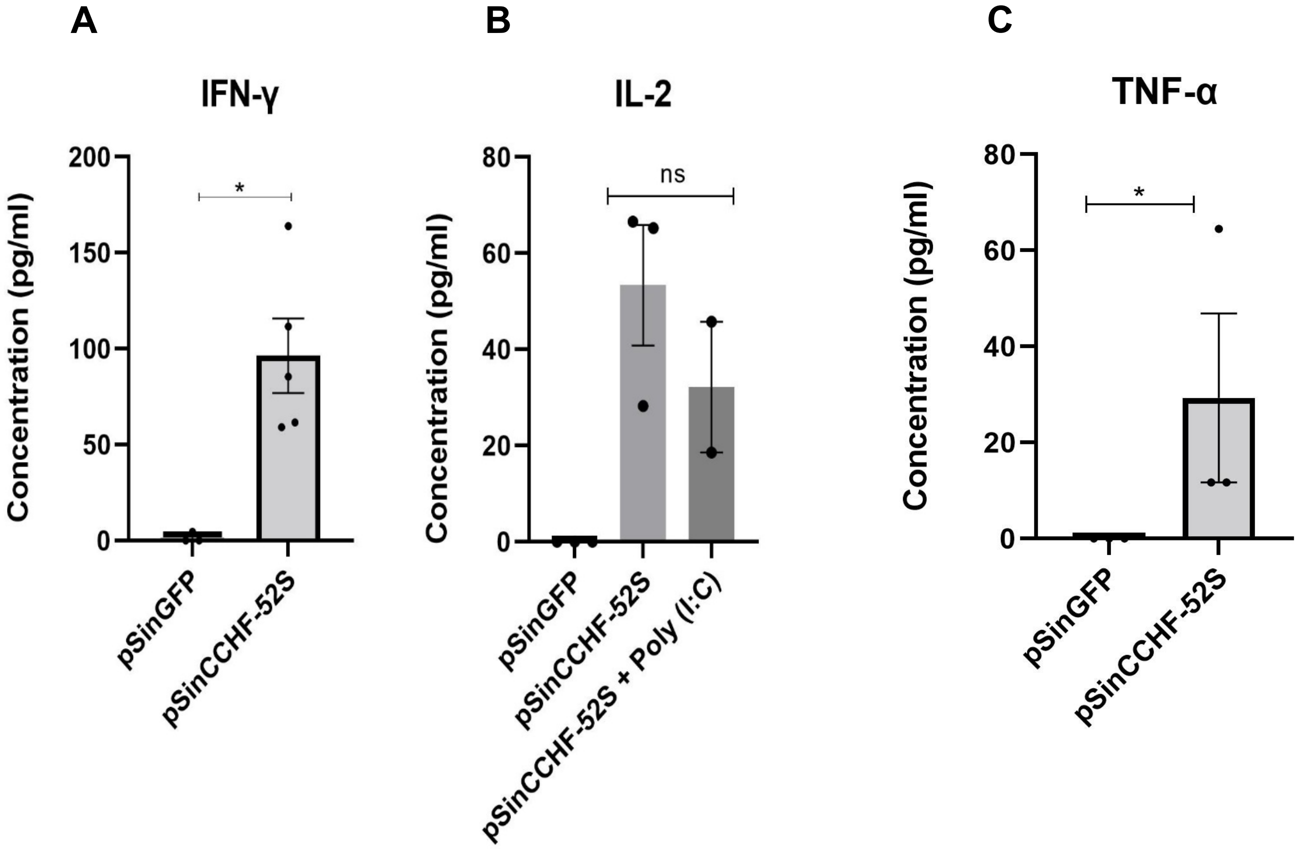

Figure S3. Cytokine profiling by ELISA from splenocytes harvested from NIH mice after immunization with pSinCCHF-52S independently (n = 3) or with adjuvant poly (I:C) (n = 2). (**A**) IFN-γ, (**B**) IL-2, (**C**) TNF-α. Data are expressed as the mean for three mice (except for pSinCCHF-52S + Poly (I:C), n = 2/group) and the standard error of the mean. * $p < 0.05$.

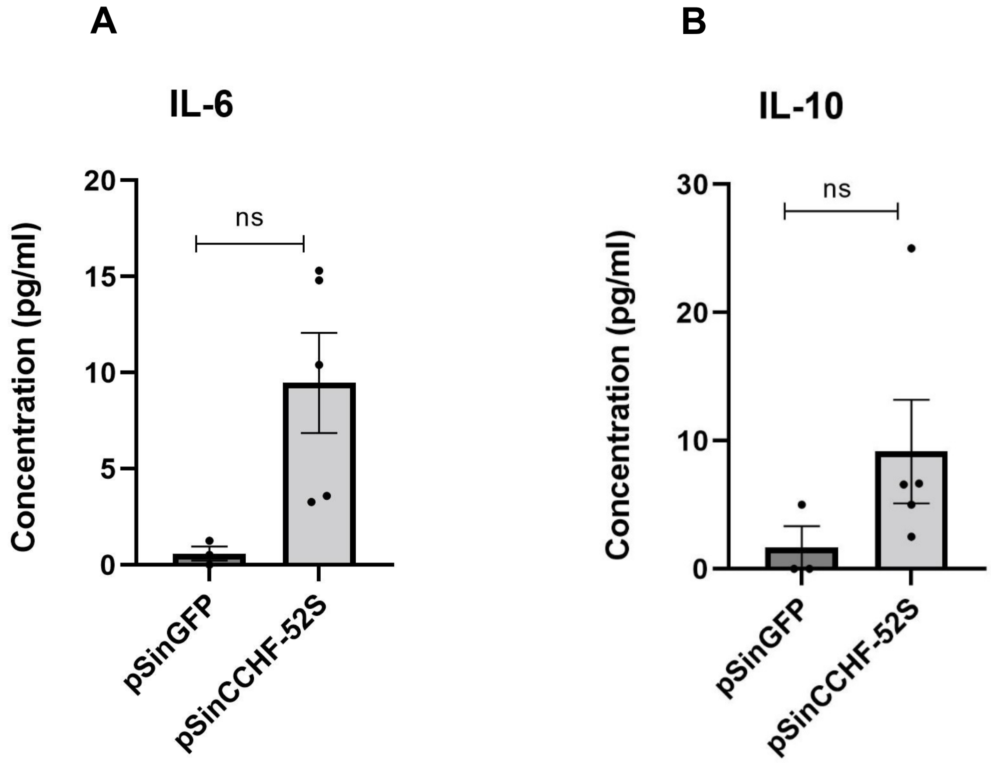

Figure S4. Cytokine profiling by ELISA from splenocytes harvested from NIH mice (n = 5/group) after immunization with pSinCCHF-52S independently or with adjuvant poly (I:C). (**A**) IL-6, (**B**) IL-10. Data are expressed as the mean for three mice (except for pSinGFP, n = 3/group) and the standard error of the mean. * $p < 0.05$.

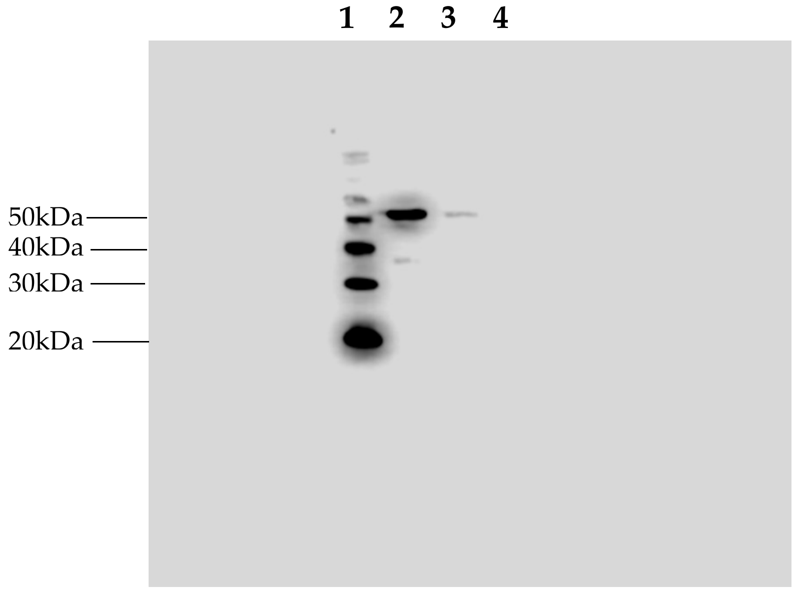

Figure S5: Western blot analysis of CCHFV NP. From left to right; Lane 1: MagicMark XP Western Protein Standard, lane 2 BHK-21 cells transfected with pSinCCHF-52S, Lane 3 BHK-21 cells transfected with pSinCCHF-31S (Replicon kinetics and immunogenicity data not reported in this study), Lane 4 Mock-transfected BHK-21 cells.
